# Research hotspots and trend of glioblastoma immunotherapy: a bibliometric and visual analysis

**DOI:** 10.3389/fonc.2024.1361530

**Published:** 2024-08-07

**Authors:** Keren Lv, Xue Du, Chunbao Chen, Yina Yu

**Affiliations:** ^1^ Department of Hematology, The First Affiliated Hospital of Zhejiang Chinese Medical University, Hangzhou, Zhejiang, China; ^2^ Yaan People’s Hospital, Sichuan University West China Hospital Yaan Hospital, Yaan, Sichuan, China; ^3^ Chengdu Pidu District People's Hospital, the 3rd Affiliated Hospital of Chengdu Medical College, Chengdu, Sichuan, China

**Keywords:** glioblastoma, immunotherapy, bibliometrics, CiteSpace, research hotspots

## Abstract

**Background:**

Glioblastoma (GBM) is one of the common malignant tumors of the central nervous system (CNS), characterized by rapid proliferation, heterogeneity, aggressiveness, proneness to recurrence after surgery, and poor prognosis. There is increasing evidence that tumorigenesis is inextricably linked to immune escape, and immunotherapy is undoubtedly an important complement to clinical treatment options for GBM, and will be a focus and hot topic in GBM treatment research. The purpose of this study was to visualize and analyze the scientific results and research trends of immunotherapy for GBM.

**Methods:**

Publications concerning immunotherapy for GBM were retrieved from the Web of Science Core Collection (WOScc) database. Bibliometric and visual analysis was performed mainly using CiteSpace and R software, and the Online Analysis Platform of Literature Metrology (https://bibliometric.com/app) for countries/regions, authors, journals, references and keywords related to publications in the field.

**Results:**

Among totally 3491 publications retrieved in this field, 1613 publications were finally obtained according to the screening criteria, including 1007 articles (62.43%) and 606 reviews (37.57%). The number of publications increased year by year, with an average growth rate (AGR) of 17.41%. Such a number was the largest in the USA (717, 44.45%), followed by China (283, 17.55%), and the USA showed the strongest international collaboration. Among the research institutions, Duke Univ (94, 5.83%) was the largest publisher in the field, followed by Harvard Med Sch (70, 4.34%). In addition, the most prolific authors in this field were OHN H SAMPSON (51) and MICHAEL LIM (43), and the degree of collaboration (DC) between authors was 98.26%. Among the co-cited authors, STUPP R (805) was the most cited author, followed by REARDON DA (448). The journal with the most published publications was FRONTIERS IN IMMUNOLOGY (75), and the most cited journal in terms of co-citation was CLIN CANCER RES (1322), followed by CANCER RES (1230). The high-frequency keyword included glioblastoma (672) and immunotherapy (377). Cluster analysis was performed on the basis of keyword co-occurrence analysis, yielding 17 clusters, based on which the current research status and future trends in the field of immunotherapy for GBM were identified.

**Conclusion:**

Immunotherapy is currently a novel treatment strategy for GBM that has attracted much attention. In the future, it is necessary to strengthen cooperation and exchanges between countries and institutions towards relevant research to promote the development of this field. Immunotherapy is expected to be an important part of the future treatment strategy for GBM, and it has already become a hot spot of current research and will be the key focus of future research.

## 1 Introduction

Glioma is the most common primary intracranial malignant tumor arising in adults, and it is difficult to be cured by surgical resection alone. Even treated by surgical resection combined with radiotherapy and chemotherapy, some tumor patients still display a poor prognosis and a high recurrence rate (1). Among all types of gliomas, GBM exhibits the highest degree of malignancy, with a 5-year survival rate of only 5%. In addition, GBM is characterized by high cellular and molecular heterogeneity, and stronger proliferation and invasion abilities (2). The last decade has witnessed dramatic progress in immunotherapy in the treatment of many solid tumors. In particular, immune checkpoint inhibitors (ICIs) have achieved satisfactory results in the treatment of solid tumors such as melanoma (3) and non-small cell lung cancer (NSCLC) (4). ICI molecules such as cytotoxic T-lymphocyte antigen-4 (CTLA-4) and programmed death receptor-1 (PD-1) have been approved for the treatment of various types of cancers and unprecedentedly prolonged the survival of patients (5). In the past, immunosuppressive cells within the glioma tumor microenvironment (TME) were thought to prevent immunotherapy from functioning (6), and the blood-brain barrier (BBB), serving as a physical and biochemical barrier, prevented therapeutic agents from entering the intracranial region (7). In addition, the central nervous system (CNS) has generally been considered an “immunologically privileged” site, which limits the effectiveness of immunotherapy for GBM.

The BBB, an important communication interface between the brain and the rest of the body, has long been thought to play a role in neurological disorders. Girolamo F et al. (8) reported the abnormal functions of forebrain pericytes during angiogenesis and barrier genesis and loss of BBB integrity directly contributes to the development of a variety of diseases. Previous studies have demonstrated that metabolic overload and associated systemic hypo-inflammation directly compromise BBB integrity by increasing paracellular permeability and decreasing trans-endothelial electrical resistance (TEER) to impair BBB function (9), suggesting that the cerebral vascular system is also an important pathological target. McArthur S et al. (10) proposed that treatment with Annexin A1 (ANXA1), a major regulator of BBB integrity and function, could be an effective therapeutic strategy. Therefore, focusing on the role of ANXA1 in the vasculature of the CNS may facilitate an in-depth understanding and the development of new therapeutic options. P-glycoprotein (P-gp) is a 170-kDa transmembrane glycoprotein that acts as an efflux pump and confers multidrug resistance (MDR) in normal tissues and tumors, including neural tissues and brain tumors. Tumor perivascular astrocytes may dedifferentiate and restore progenitor-like P-gp activity to become MDR cells, contributing to the MDR profile of GBM vessels together with perivascular P-gp expressing glioma stem like cells (GSCs) (11). Moreover, multiple cellular sources in the GBM vasculature may be associated with P-gp-mediated chemoresistance and may be accountable for GBM treatment failure and tumor recurrence. In addition, microglia not only appear in and around brain tumors but also contribute significantly to the actual tumor mass (12), with evidence that the behavior of microglia is controlled by tumor cells, supporting their growth and infiltration. Recent data demonstrate that neurons synapse directly onto glioma cells and drive their proliferation and spread through glutamatergic action. Microglia, as CNS-resident myeloid cells, can regulate glioma growth, prune synapses and promote synapse formation (8). Errede M et al. (13) by analyzing the cellular origin of chemokine CCL2, a molecule involved in immune cell recruitment and BBB-microvascular leakage, showedan increase in microglia is the hallmark of encephalomyelitis (EAE) in the mouse neocortex, which is characterized by a high CCL2 expression level. Therefore, targeting molecules in the GBM microenvironment and the oncogenic activity of microglia can help manage and slow down the growth of refractory high-grade gliomas.In recent years, it has been found that the CNS can deliver antigens through various pathways such as lymphatic reflux and crossing the BBB, and recruit immune cells into brain tissues and tumors in the event of gliomas, indicating that gliomas have a physiological structural basis for immunotherapy (14). The meninges, the plasma membrane structures surrounding the CNS, and contain a wide reservoir of immune cells, and the meningeal lymphatics are a key pathway for cerebrospinal fluid (CSF) to enter the peripheral blood, where they provide immune surveillance of brain tissues (15, 16). The brain is connected to the peripheral immune system through the meningeal lymphatics (17), and this important finding makes immunotherapy the most promising therapeutic strategy for GBM. Recently, the presence of lymph node-like structures, called tertiary lymphoid structures (TLS), has been confirmed in patients with gliomas, but not in healthy individuals (18). TLS contain all the components needed to support on-site lymphocyte activation, which implies that they may positively influence the anti-tumor immune response. In addition, it has been revealed that immunotherapy can regulate the formation of TLS in the brain, providing exciting opportunities to find new ways to regulate the anti-tumor immune response in gliomas. With a comprehensive delineation of the unique immunobiology of gliomas, immunotherapy for gliomas will be fundamentally reshaped.

At present, immunotherapy is the focus of numerous preclinical studies and clinical trials on GBM are focused on. ICIs include PD-1, PD-L1, and CTLA-4 inhibitors, and preclinical studies have shown their promise in the treatment of GBM (19). In a randomized, multi-institutional clinical trial, 35 patients with recurrent, surgically resectable GBM, patients who were randomized to receive neoadjuvant pembrolizumab followed by postoperative adjuvant pembrolizumab therapy had an extended median overall survival (OS) compared those who received postoperative adjuvant pembrolizumab therapy alone (14 months vs. 7.6 months), and OS also showed the same trend (20). This result indicates that neoadjuvant administration of PD-1 blockade enhances local and systemic antitumor immune responses and may represent a more effective treatment for this lethal brain tumor. In June 2020, the Food and Drug Administration (FDA) approved pembrolizumab for the pan-cancer treatment of patients with solid tumors including gliomas of the CNS, showing high tumor mutation burden (TMB-high), defined as ≥ 10 mutations per megabase (mut/mb) (21),. A phase I clinical trial on recurrent GBM (rGBM) confirmed that perioperative intravenous administration of ipilimumab (IPI, a CTLA-4 inhibitor) ± nivolumab (NIVO, a PD-1 inhibitor) in rGBM was safe, and exploratory findings merit further investigation of immunotherapy for GBM (22). Rindopepimut (also known as CDX-110), a vaccine targeting the epidermal growth factor receptor (EGFR) deletion mutation EGFRvIII, has received drug approval from the US FDA for the breakthrough treatment of EGFRvIII-positive gliomas in adult patients (23). In an open-label, first-in-human trial evaluating the safety and therapeutic potential of cytomegalovirus-specific (CMV-specific) adoptive cellular therapy (ACT) in the adjuvant treatment of patients with primary GBM, the data obtained suggest that CMV-specific ACT can be a safe adjuvant therapy for primary GBM and, if performed before recurrence, this therapy may improve OS of GBM patients (24). Intratumoral infusion of nonpathogenic polio-rhinovirus chimera (PVSRIPO) in patients with rGBMdemonstrated no potential neurotoxicity. Patients receiving PVSRIPO immunotherapy had higher survival rates at 24 and 36 months than historical controls (25). Although some small-scale studies have reported that glioma patients can benefit from immunotherapy to varying degrees, there remain challenges requiring more in-depth studies and clinical trials. The rapidly developing immunomics, genomics, sequencing technologies, and ICIs have created new opportunities for immunotherapy, one of the important adjuvant therapies for gliomas. Meanwhile, the evolving nanotechnology (26) enable the targeting of tumor sites across the BBB, which may also bring new possibilities for immunotherapy for gliomas.

Bibliometrics is the discipline that applies mathematical and statistical methods to the study of books and other communication media (27), allowing for the qualitative and quantitative evaluation of trends in literature research. Bibliometric methods and tools can be used to analyze a larger volume of literature data from a more macroscopic perspective in order to accurately grasp the development trend and research hotspots in a field and provide reference for the research of researchers in related fields. This study uses bibliometric tools to measure and visualize the publications in this field in the last decade in order to understand the current status and trends of research in this field and to provide a scientific reference for the researchers working on immunotherapy for GBM.

## 2 Materials and methods

### 2.1 Data source

**Figure 1:**
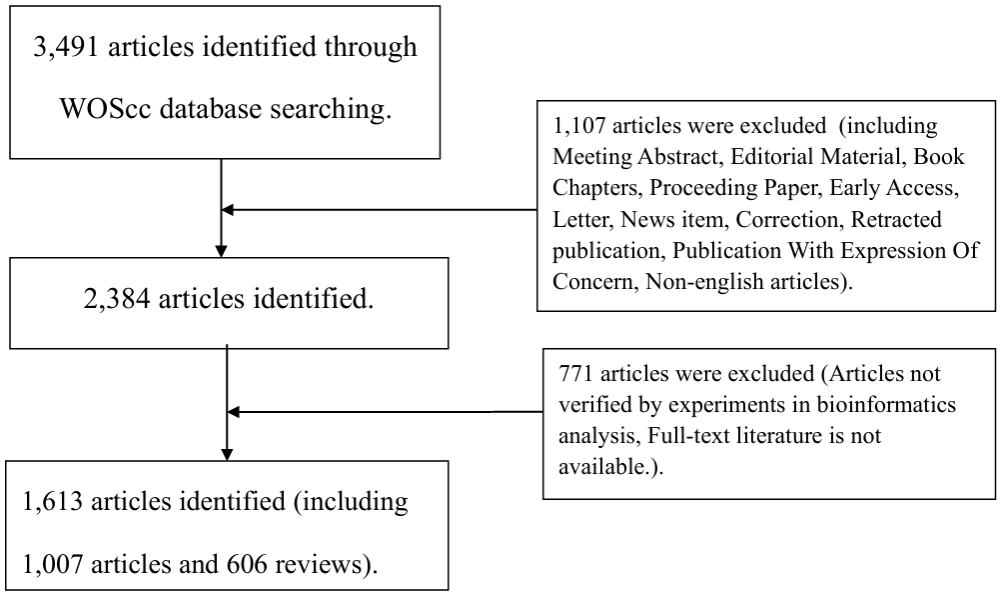
Flowchart of the literature selection process.

The Web of Science Core Collection (WOScc) online database is an important database for global access to scholarly journals and considered to be the best database for bibliometric analysis (28). In July 2022, literature related to immunotherapy for GBM was searched on WOScc, with the time span from 2012 to July 2022. The search strategy was: TS = (glioblastoma* OR “glioblastoma multiform*” OR “ malignant glioma” OR “brain cancer” OR gliosarcoma OR spongioblastoma) AND TS = (Immunotherapy OR Immunotherapies OR immunotherapeutic). Literature inclusion criteria: (1) literature with immunotherapy for GBM as the research topic; (2) articles and reviews; (3) literature published in English. Literature exclusion criteria: (1) Literature irrelevant to immunotherapy for GBM; (2) conference abstracts, news, case studies, bioinformatic analysis without experimental validation, etc.; (3) literature that was not available in full-text format. To ensure the quality of the search, the literature obtained was evaluated by two reviewers, and any disagreements were resolved through discussion until consensus was reached. Flowchart of the literature selection process is shown in **Figure 1**.

### 2.2 Analysis method

Bibliometrics analysis and visualization of literature in related fields were performed primarily through CiteSpace (version 5.8.R3) and R (version 4.1.3) software, as well as the online analysis platform of literature metrology (https://bibliometric.com/app), using Microsoft Excel 2019 for data management. CiteSpace software, developed by Prof. Chaomei Chen as a literature visualization tool, was designed to mine literature data and visualize the evolution of a knowledge domain in the form of a map (29). Betweenness centrality is an important parameter in CiteSpace. In general, centrality ≥ 0.1 is considered an important node, and CiteSpace will also mark it with a purple circle. The “bibliometrix” package in R software was used to visualize and analyze journals. Bibliographic data are exported from the WOS database in plain the text format, with full records and cited refercnces, 1,000 records at a time, and saved in a folder. Import the file into CiteSpace or R software through the “File”->“Import” menu to analyse and process the data and output the visualisation maps. The online analysis platform of literature metrology was developed by Chinese scholars for the integral literature analysis, partnership analysis, subject and journal analysis, keyword analysis, and citation network analysis. Moreover, the bibliometric online analysis platform was adopted to analyze country/region collaborations.

### 2.3 Statistical analysis

Statistical data were processed using SPSS (IBM SPSS Statistics 27). *p*<0.05 indicated a statistically significant difference.

## 3 Results

### 3.1 Publishing trend

**Figure 2:**
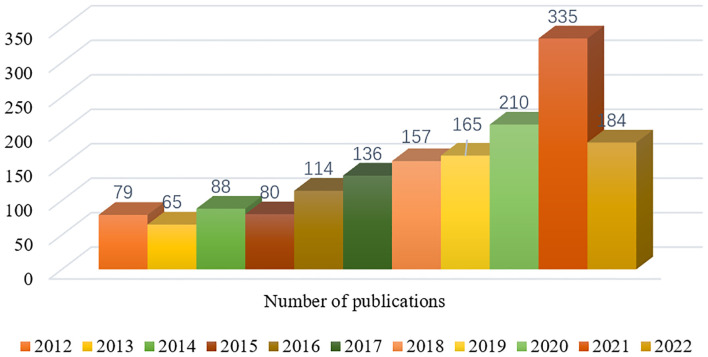
Annual number of publications in the relevant literature from 2012 to July 2022.

A total of 3491 publications concerning immunotherapy for GBM published from 2012 to July 2022 were retrieved from WOScc, and 1613 articles (62.43%) and 606 reviews (37.57%) were finally filtered based on the set search criteria. **Figure 2** shows the annual publication volume from 2012 to July 2022. The number of publications was almost stable from 2012 to 2015, with a steady increase from 2016 to 2021. The annual publications exceeded 200 in 2019 and 335 in 2020, and the average growth rate (AGR) was 17.41% from 2012 to 2021. Furthermore, the compound annual growth rate (CAGR) of publications (30) gradually increased from 35.01% in 2013 to 40.07% in 2016, before decreasing to 33.58% in 2021 (**Supplementary Table S1**; **Supplementary Figure S1A**). This indicates that the CAGR is basically on a downward trend, although the annual production is increasing year by year. As illustrated in **Supplementary Table S2**; **Supplementary Figure S1B**, the relative growth rate (RGR) decreased from 2013 (60.04%) to 2021 (26.71%). A direct equivalence relation existed between RGR and doubling time (DT) (30), and the DT increased from 1.15 in 2013 to 2.59 in 2021 (**Supplementary Table S2**; **Supplementary Figure S1C**). In addition, the correlation between publications and citations was determined by Pearson correlation analysis, and a p-value < 0.05 was considered as a significant correlation. The results of this analysis showed a high positive correlation between publications and citations (*r*=0.973, *p*<0.001).

### 3.2 Countries/regions and institutions

**Figure 3:**
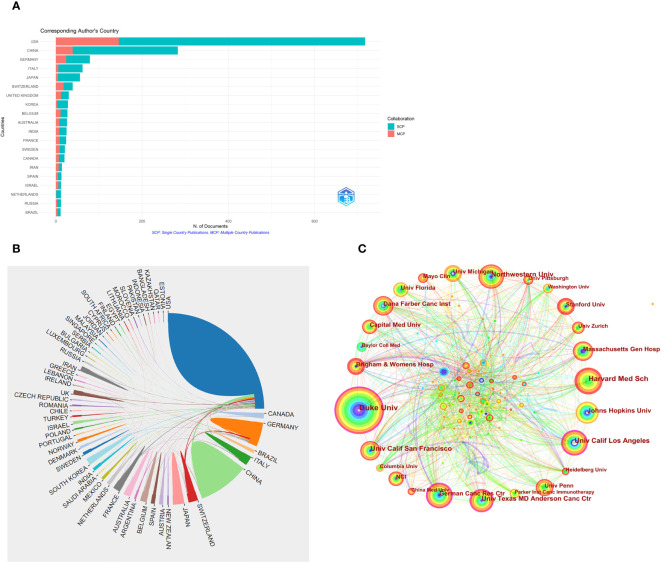
**(A)** International collaboration intensity of a country with relevant publications. **(B)** Inter-country cooperation relations for related publications. **(C)** Cooperation mapping between related publication institutions.

A total of 1613 publications from 1868 institutions in 54 different countries/regions were retrieved based on the search criteria. The number of publications was the largest in USA (717, 44.45%), followed by China (283, 17.55%), which were much higher than other countries/regions (**Supplementary Table S3**). The intensity of international cooperation of countries was analyzed using the “bibliometrix” package of the R software. Multiple country publications (MCP) refer to publications with at least one co-author from a different country, while single country publications (SCP) refer to publications with co-authors from a single country. The largest number of MCP was from USA (146), with an MCP-Ratio of 20.36%, followed by China (39) with an MCP-Ratio of 13.49% (**Supplementary Table S3**; **Figure 3A**), indicating that USA has the highest intensity of international cooperation, followed by China. Further, online analysis platform of literature metrology was used to visualize the inter-country cooperation. **Figure 3B** shows the inter-country cooperation network. It was found that the USA cooperated most closely with other countries, and the countries with the most cooperation included China, South Korea and Switzerland, followed by Germany.

The research institution with the most publications was Duke Univ (94 articles, 5.83%), followed by Harvard Med Sch (70 articles, 4.34%). Among these institutions, Duke Univ and Univ Calif Los Angeles showed the highest centrality (0.13), followed by Univ Texas MD Anderson Canc Ctr and German Canc Res Ctr, with the centrality of 0.11 (**Table 1**). In the CiteSpace visualization atlas, each circle represents an institution, the size of the circle indicates the number of publications of the institution, the connecting lines between the circles indicate the cooperation between institutions, the nodes with high centrality are shown as purple rings, and the thickness of the purple rings depicts the value of centrality in size (**Figure 3C**).

**Table 1:** Top 10 institutions for related publications.

Rank	Count	Centrality	Institutions
1	94	0.13	Duke Univ
2	70	0.08	Harvard Med Sch
3	53	0.04	Northwestern Univ
4	53	0.13	Univ Calif Los Angeles
5	50	0.09	Univ Calif San Francisco
6	45	0.06	Johns Hopkins Univ
7	42	0.11	Univ Texas MD Anderson Canc Ctr
8	40	0.06	Dana Farber Canc Inst
9	39	0.11	German Canc Res Ctr
10	38	0.03	Massachusetts Gen Hosp

### 3.3 Authors and co-cited authors

**Figure 4:**
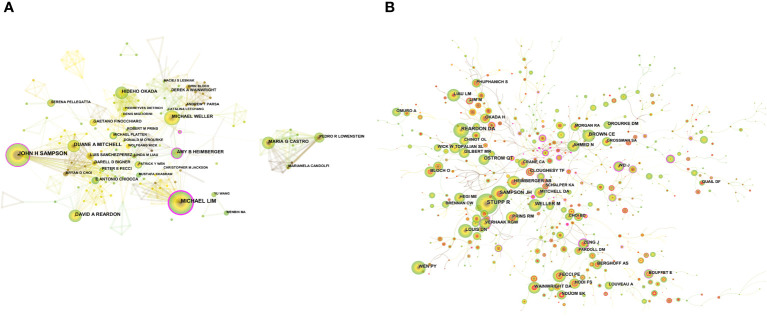
**(A)** CiteSpace visualization maps of the authors of related publications. **(B)** CiteSpace visualization maps of co-cited authors of related publications.

A total of 8503 researchers participated in the publication of the relevant literature. JOHN H SAMPSON (51) and MICHAEL LIM (43) published the largest number of publications, followed by DAVID A REARDON (29), DUANE A MITCHELL (27) and HIDEHO OKADA (25). The top 10 authors had the highest centrality in MICHAEL LIM (0.21), followed by AMY B HEIMBERGER (0.18) (**Supplementary Table S4**). The degree of cooperation (DC) between authors was 98.26% (30). **Figure 4A** shows the visual analysis map of author cooperation network, where each circle node represents an author (the larger the node, the more the publications), the line between the nodes represents the connection between authors (the thicker the line, the closer the cooperation), and the purple circle outside the node marks the higher centrality.

The co-citation of documents was previously used as a measure of the relatedness between documents. Later, co-citation was introduced to the author dimension, and the method of author co-citation analysis (ACA) was developed, by which a co-citation relationship constituted by two or more authors cited by one or more publications at the same time can be analyzed (31). Among the co-cited authors, STUPP R (805) was the most cited author, followed by REARDON DA (448), SAMPSON JH (432). FECCI PE (0.1) had the highest centrality among the top ten co-cited authors (**Supplementary Table S4**). A clinical study led by Professor Stupp demonstrated that temozolomide (TMZ) combined with radiotherapy prolonged the survival of adult GBM patients, and the Stupp protocol of simultaneous radiotherapy for gliomas, named after the professor, was then widely used in the clinic to date (32). **Figure 4B** shows a visual network map of the relationship between co-cited authors.

### 3.4 Journals and co-cited journals

**Figure 5:**
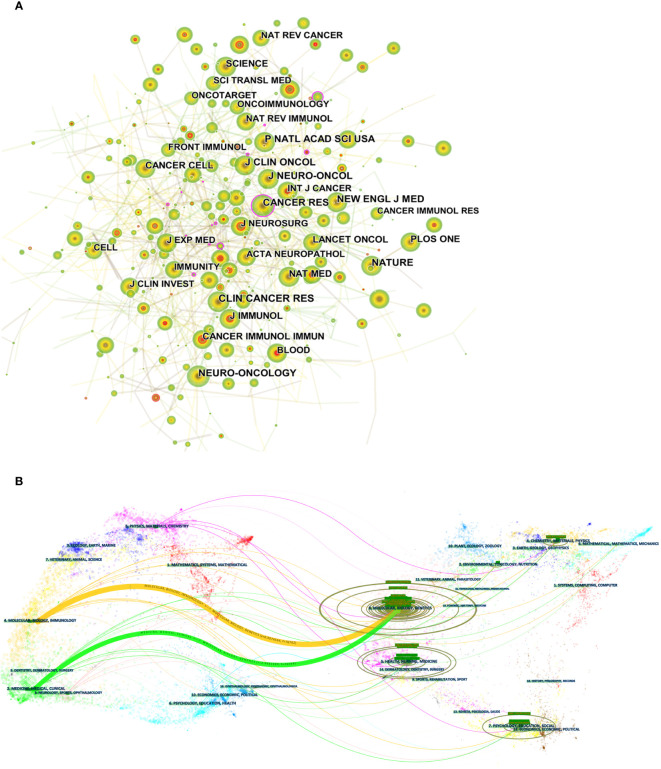
**(A)** CiteSpace visualization maps of co-cited journals of related publications. **(B)** The dual-map overlay of journals of related publications.

The “bibliometrix” package in R software was used to visualize and analyze the source journals of the publications. FRONTIERS IN IMMUNOLOGY was the journal with the largest number of publications (75), followed by JOURNAL OF NEURO-ONCOLOGY (58). As shown in **Supplementary Figure S2A**, 7 journals had more than 50 publications. Among the top 10 academic journals, CLINICAL CANCER RESEARCH (13.801) displayed the highest impact factor (IF) (**Table 2**). Among the 826 co-cited journals, the most cited journal was CLIN CANCER RES (1322), followed by CANCER RES (1230) (**Table 3**). CANCER RES had the highest centrality (0.2), indicating its high influence in this research field. Journal co-citation reflects the correlation between various journals and disciplines. **Figure 5A** shows the CiteSpace visualization map of co-cited journals, where the size of the circle represents the frequency of co-citation, and the purple circle indicates the higher centrality.

**Table 2:** Top 10 source journals for related publications.

Rank	Count	Sources Journal	IF 2022	JCR
1	75	FRONTIERS IN IMMUNOLOGY	8.786	Q1
2	58	JOURNAL OF NEURO-ONCOLOGY	4.506	Q2
3	56	CLINICAL CANCER RESEARCH	13.801	Q1
4	56	FRONTIERS IN ONCOLOGY	5.738	Q2
5	56	NEURO-ONCOLOGY	13.029	Q1
6	55	CANCERS	6.575	Q1
7	50	ONCOIMMUNOLOGY	7.723	Q1
8	37	CANCER IMMUNOLOGY IMMUNOTHERAPY	6.63	Q1
9	33	INTERNATIONAL JOURNAL OF MOLECULAR SCIENCES	6.208	Q1
10	27	JOURNAL FOR IMMUNOTHERAPY OF CANCER	12.47	Q1

**Table 3:** Top 10 co-cited journals of related publications.

Rank	Count	Centrality	Co-cited Journals	IF2022	JCR
1	1322	0.05	CLIN CANCER RES	13.801	Q1
2	1230	0.2	CANCER RES	13.312	Q1
3	1216	0	NEURO-ONCOLOGY	13.029	Q1
4	1148	0	NEW ENGL J MED	176.079	Q1
5	995	0.1	NATURE	69.504	Q1
6	960	0.09	J CLIN ONCOL	50.717	Q1
7	949	0.08	J NEURO-ONCOL	4.506	Q2
8	934	0.08	P NATL ACAD SCI USA	12.779	Q1
9	876	0	NAT MED	87.241	Q1
10	838	0	PLOS ONE	3.752	Q2

The dual-map overlay of journals displays information about the distribution, citation trajectory, and shift of gravity of publications across disciplines (33). The left side shows the distribution of citing journals, while the right side displays the distribution of cited journals. In **Figure 5B**, the colored paths indicate the cited relationships. Among them, the yellow paths indicate that literature published in molecular/biology/immunology journals is frequently cited by molecular/biology/genetics journals, and green paths indicate that literature published in molecular/medical/clinical journals is frequently cited in molecular/biological/genetics journals.

### 3.5 Co-cited references and citation burst analysis

**Figure 6:**
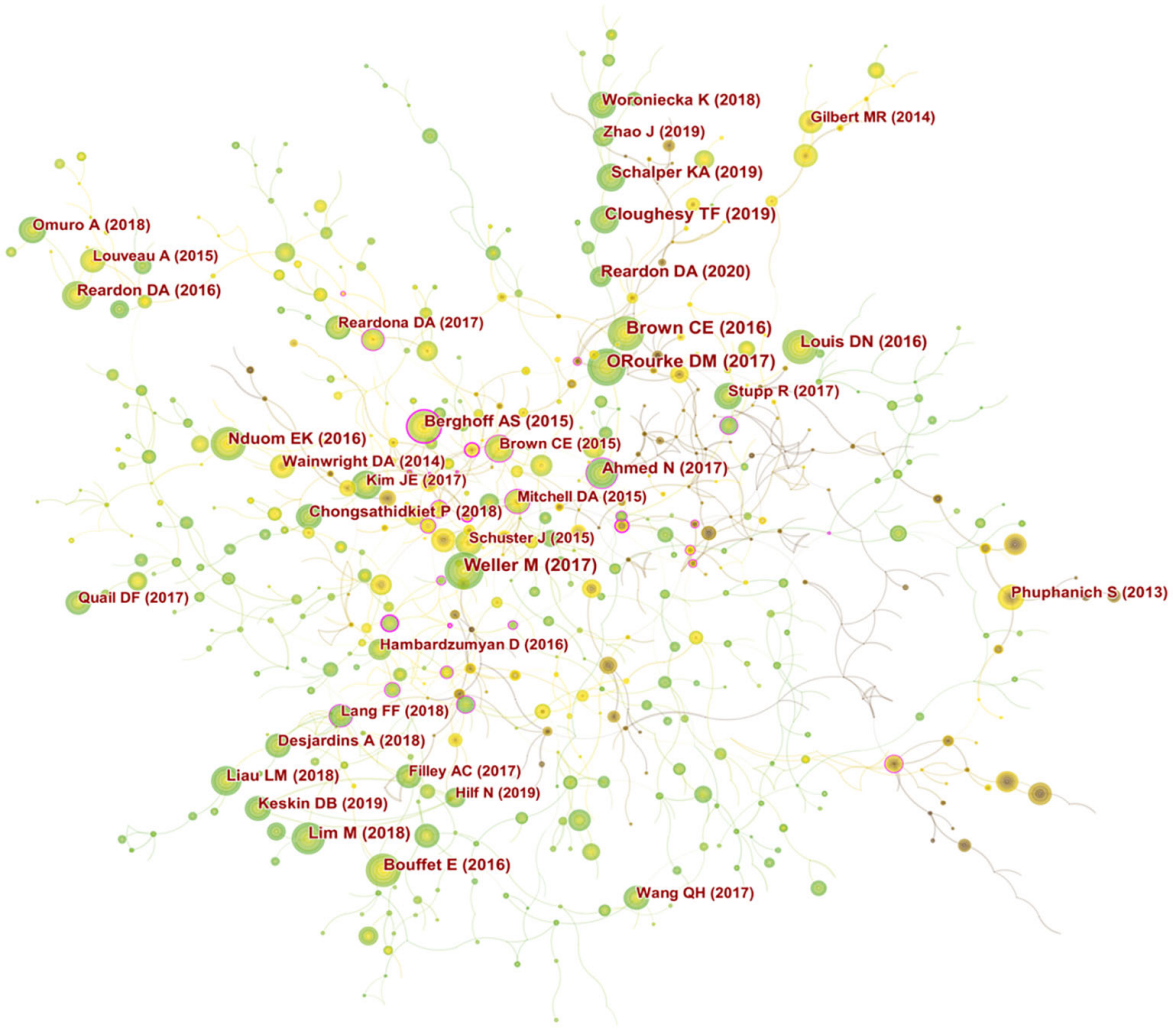
Visualization mapping of co-cited references of related publications.

**Table 4** lists the top 10 co-cited references out of 983 co-cited references. **Figure 6** shows a visualization of the co-cited references. O’Rourke DM et al. (34) published the article ‘‘A single dose of peripherally infused EGFRvIII-directed CAR T cells mediates antigen loss and induces adaptive resistance in patients with recurrent glioblastoma’’, which was the most frequently cited article (203), reporting a first-in-human study of intravenous delivery of a single dose of autologous T cells redirected to the EGFRvIII mutation by a chimeric antigen receptor (CAR). The results revealed that the treatment for the first 10 patients with GBM showed feasibility and safety. In addition, ‘‘Regression of Glioblastoma after Chimeric Antigen Receptor T-Cell Therapy’’, the study of Brown CE et al. (35) evaluated the role of intracranial CAR T-cell therapy targeting interleukin-13 receptor alpha 2 (IL13Rα2) in patients with malignant gliomas. This study provides initial evidence for the safety and antitumor activity of CAR T-cell immunotherapy in patients with malignant brain tumors. Moreover, the titles of the top 10 co-cited references, demonstrated that the corresponding research involved CAR T-Cell Therapy, neoadjuvant immunotherapy, randomized clinical trials, immunotherapy for rGBM, and ICIs.

**Table 4:** Top 10 co-cited references of related publications.

Rank	Count	Centrality	Year	Co-Cited References
1	203	0.07	2017	A single dose of peripherally infused EGFRvIII-directed CAR T cells mediates antigen loss and induces adaptive resistance in patients with recurrent glioblastoma
2	200	0	2016	Regression of Glioblastoma after Chimeric Antigen Receptor T-Cell Therapy
3	186	0	2017	Rindopepimut with temozolomide for patients with newly diagnosed, EGFRvIII-expressing glioblastoma (ACT IV): a randomised, double-blind, international phase 3 trial
4	180	0.05	2019	Neoadjuvant anti-PD-1 immunotherapy promotes a survival benefit with intratumoral and systemic immune responses in recurrent glioblastoma
5	153	0	2016	The 2016 World Health Organization Classification of Tumors of the Central Nervous System: a summary
6	152	0.01	2018	Current state of immunotherapy for glioblastoma
7	143	0.06	2020	Effect of Nivolumab vs Bevacizumab in Patients With Recurrent Glioblastoma The CheckMate 143 Phase 3 Randomized Clinical Trial
8	129	0.01	2016	Immune Checkpoint Inhibition for Hypermutant Glioblastoma Multiforme Resulting From Germline Biallelic Mismatch Repair Deficiency
9	124	0.15	2017	HER2-Specific Chimeric Antigen Receptor-Modified Virus-Specific T Cells for Progressive Glioblastoma A Phase 1 Dose-Escalation Trial
10	124	0.05	2019	Neoadjuvant nivolumab modifies the tumor immune microenvironment in resectable glioblastoma

Citation burst analysis identifies literature that has been of interest to researchers in related fields over time. According to the strongest citation burst (**Supplementary Figure S3**), the first citation burst started in 2012, with a citation burst strength of 16.99-40.52 for the first 25 references. Among them, the reference with the strongest citation burst was published by Sampson JH et al. (36) in the journal “J Clin Oncol”. This phase II, multicenter, prospective trial was conducted to assess the immunogenicity of an EGFRvIII-targeted peptide vaccine and to estimate progression-free survival (PFS) from vaccination and histologic diagnosis in patients newly diagnosed with GBM who expressed EGFRvIII. The findings of this trial warrant investigation in a phase III randomized trial. The two references with the citation burst from 2020 to date are both studies on neoadjuvant immunotherapy for gliomas (20, 37).

### 3.6 Keyword co-occurrence clustering and time zone analysis

**Figure 7:**
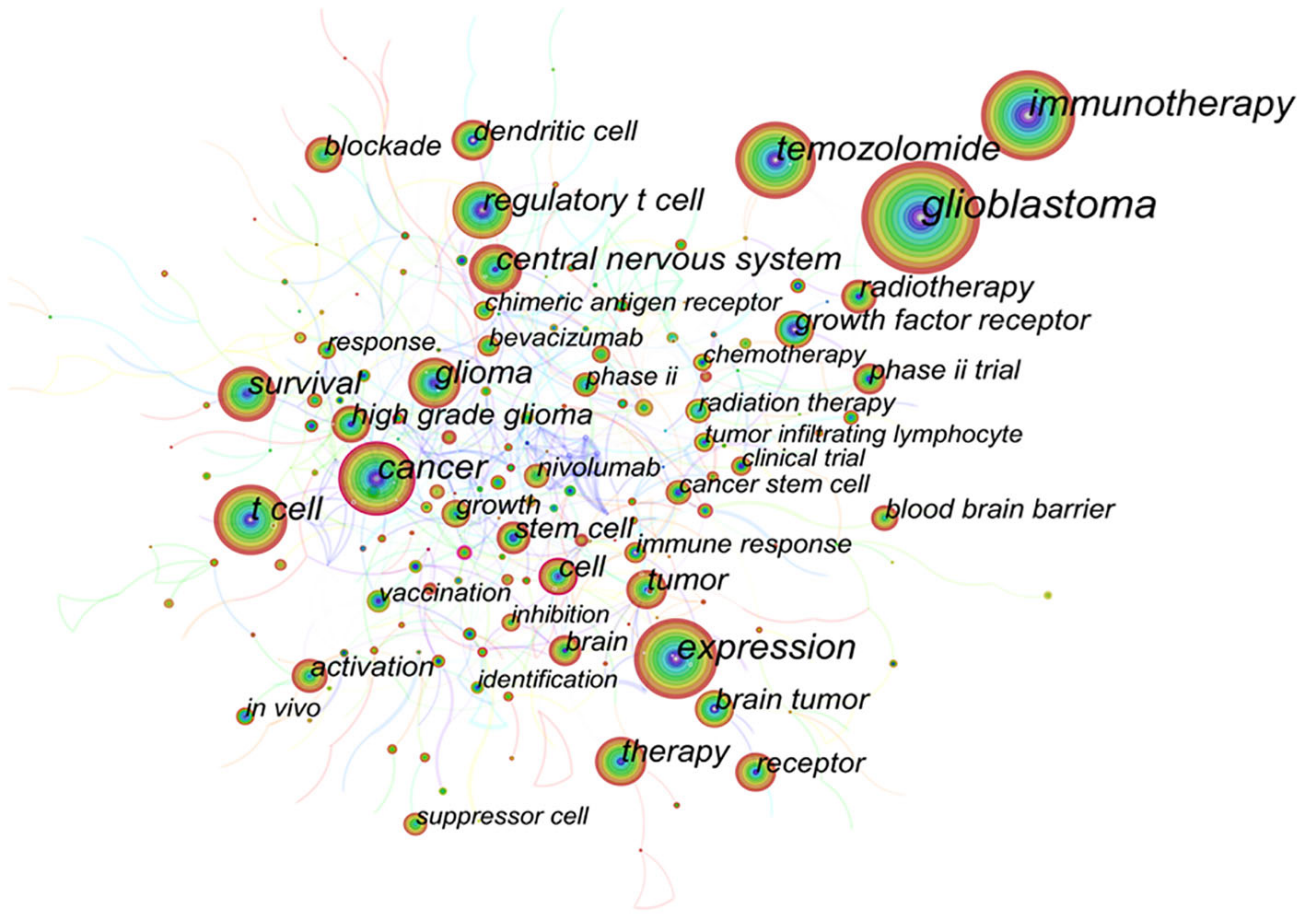
Visualization map of keyword co-occurrence for related publications.

Keywords are the subject of research content, and the co-occurrence analysis of keywords can summarize the research hotspots in a specific field. **Supplementary Table S5** shows the top 10 keywords that emerged frequently in studies related to immunotherapy for GBM, including glioblastoma (672), immunotherapy (377), expression (322), temozolomide (273), cancer (261), T cell (212), survival (178), glioma (173), central nervous system (167), and regulatory T cell (152), indicating that they are the current research hotspots related to immunotherapy for GBM. In the visualization map of keyword co-occurrence, there are 461 nodes and 775 lines (**Figure 7**). Each node corresponded to a keyword, and larger nodes indicated higher frequency. Besides, the number of links between nodes and distance between nodes reflected the tightness between keywords. Further clustering analysis of keywords based on keyword co-occurrence analysis reflected the hot research directions in this field. **Supplementary Figure S4A** shows the visualization map of the clustering of keywords, mainly including regulatory T cell, macrophage, peptide vaccination, PD-1, CD8(+), chimeric antigen receptor, innate immunity, phase II trial, tumor microenvironment, tumor heterogeneity, immunotherapy, IDH mutation, glioma, natural killer cell, survival, and resistance 17 clusters. Furthermore, keyword time zone map analysis was conducted to reveal the keywords as time zones according to the time when the keywords first appeared, further showing the time zone evolution of the keywords (**Supplementary Figure S4B**).

Keyword burst analysis reveals the phase and duration of research hotspots in a field. Herein, the keyword burst map showed the burst strength of the top 25 keywords (**Supplementary Figure S4C**), where the blue line indicated the time line, and the red part of the blue time line indicated the burst duration. Among the 25 keywords, nivolumab (6.76) had the highest burst strength, followed by recurrent glioma (6.19); antitumor immunity was the keyword with the longest burst duration; double blind and resistance were the keywords with the burst from 2020 to date.

## 4 Discussion

### 4.1 General information

Trends in publications indicate the dynamics of research in a field. Here, **Figure 2** shows that the number of GBM immunotherapy-related publications is on the rise, with an AGR of 17.41% from 2012 to 2021. The number of publications by countries/regions and research institutions provided an objective indication of the scientific level and influence of the relevant research field. The largest number of publications was from USA (717, 44.45%), followed by China (283, 17.55%), indicating that USA and China, the main scientific exporters in this research field, have contributed significantly to the development of the field. The analysis of the intensity of international cooperation and partnership networks of countries revealed that USA cooperated most closely with other countries, while there was less cooperation between other research countries. Therefore, it is necessary to strengthen the research cooperation between countries and enhance international exchange in future research to promote the development of the field. Among the 8503 researchers, JOHN H SAMPSON published the most papers (51), followed by MICHAEL LIM (43). Among the top 10 authors, MICHAEL LIM had the highest centrality (0.21), followed by AMY B HEIMBERGER (0.18), which demonstrated their great contribution to the development of the field. Most of Sampson JH’s publications were reviews or clinical trial studies on immunotherapy for gliomas. A snapshot of the current GBM clinical trial landscape is provided in a review that offers valuable suggestions for optimizing clinical trial protocols for GBM (38). STUPP R (805) was the most cited author, and the protocol of simultaneous radiotherapy for gliomas named after Stupp is widely used in clinical practice today (32).

The IF of journals is an important indicator to evaluate the academic influence of journals. The largest number of publications appeared in FRONTIERS IN IMMUNOLOGY (75), followed by JOURNAL OF NEURO-ONCOLOGY (58), and among the top 10 academic journals, CLINICAL CANCER RESEARCH had the highest IF (13.801) (**Table 2**), showing that these journals have a certain influence in immunotherapy for GBM. A dual-map overlay of journals shows that literature published in molecular/biology/immunology journals is frequently cited in molecular/biology/genetics journals. The reference co-citation analysis identifies important publications that form part of the research clustering themes in related fields. According to the top 10 co-cited references, the main research directions in the field included CAR T-Cell Therapy, neoadjuvant immunotherapy, randomized clinical trials, immunotherapy for rGBM, and ICIs. The reference with the highest citation burst strength was reported by Sampson JH et al. (36), which was a phase II, multicenter, prospective trial conducted to assess the immunogenicity of an EGFRvIII-targeted peptide vaccine and to estimate progression-free survival (PFS) from vaccination and histologic diagnosis in patients newly diagnosed with GBM who expressed EGFRvIII. The two references with the citation burst to date are studies on neoadjuvant immunotherapy for gliomas.

### 4.2 Research hotspots

Keywords represent the content of the literature, and the analysis of the frequency of keywords directly reflects the research hotspots and development trends in a certain subject field. The top 10 high-frequency keywords from publications related to immunotherapy for GBM included glioblastoma (672), immunotherapy (377), expression (322), temozolomide (273), cancer (261), T cell (212), survival (178), glioma (173), central nervous system (167), and regulatory T cell (152). Cluster analysis was performed based on keyword co-occurrence analysis, and 17 clusters were finally established, mainly including regulatory T cell, macrophage, peptide vaccination, PD-1, CD8(+), chimeric antigen receptor, innate immunity, phase II trial, tumor microenvironment, tumor heterogeneity, immunotherapy, IDH mutation, glioma, natural killer cell, survival, and resistance. This analysis identified the current research hotspots and possible future trends in immunotherapy for GBM. The main contents are as follows:

#### 4.2.1 Possibility of immunotherapy for GBM

Immunotherapy is treatment that uses a patient’s immune system fight malignant tumors. In 1891, William B. Coley, a bone sarcoma surgeon, carried out the study of immunotherapy for the treatment of malignant tumors for the first time. Coley injected streptococcal organisms into a cancer patient to cause erysipelas and stimulate the immune system. The patient’s tumor disappeared, presumably because of an attack by the immune system. Since then, Coley began the lifelong study of immunotherapy, later making him known as the ‘‘Father of Immunotherapy’’ (39). However, the CNS had long been regarded as an immune privileged system for many years, referring to the lack of specialized lymphatic channels in the brain. The concept of immune privilege was based on the original experimental data reported by Peter Medawar 50 years ago, which showed that foreign cells implanted in rodent brains were successfully transplanted, whereas the same cells were eliminated by the host immune system when placed in peripheral tissues (40). Until 2015, Louveau et al. (17) found functional lymphatic vessels within the dural sinus in their search for channels for T cells to enter and exit the meninges. These structures show all the molecular characteristics of lymphatic endothelial cells. They are capable of carrying both fluid and immune cells from the CSF, and connecting deep cervical lymph nodes. This finding suggests that the current dogma on brain tolerance and immune privilege is being revisited, and casts new light to the treatment of gliomas. Although the brain is an immunologically privileged site, the immune microenvironment offers the possibility of implementing immunotherapy for brain tumors. TLS are ectopic lymphoid formations arising in inflamed, infected, or tumoral tissues. They exhibit all the characteristics of structures in the lymph nodes (LN) associated with the generation of an adaptive immune response, including a T cell zone with mature dendritic cells (DC), a germinal center with follicular dendritic cells (FDC) and proliferating B cells (41). In tumors, TLS is thought to provide an alternative to tumor-draining lymph nodes as a site for antigen expression and activation of nascent T cells. Recent studies have demonstrated that the immunostimulatory agonistic CD40 antibody (αCD40) induces the formation of TLS near meningeal tissues in preclinical glioma models. Studies have revealed that the TLS is present in human gliomas and is associated with an increasing number of intratumoral T cells in GBM, suggesting an association between TLS and the regulation of immune responses in glioma patients (18). TLS in the brain can be manipulated therapeutically, probably triggering or suppressing immune responses. As immunotherapy faces many difficulties and challenges, current research continues to explore the immune basis for effective treatment of gliomas, which provides a strong basis for the development of novel therapeutic strategies in the future.

#### 4.2.2 The unique immune environment of GBM

Over the past decade, immunotherapy has dramatically changed the clinical outcome of tumors. However, this promising immunotherapy has encountered serious challenges when applied in the treatment of gliomas, mainly due to the presence of the BBB and the immunosuppressive character of the TME. The BBB serves as an important protective barrier for the brain, controlling the exchange of substances between the blood and the CNS, and maintaining homeostasis within the CNS (42). Meanwhile, the BBB also provides a physical and biochemical barrier for drugs to enter the brain. The tight junctions and adhesions between the endothelial cells of the brain capillaries prevent intercellular diffusion, and molecules from the blood can only enter the brain through the luminal and plasma membranes of the endothelial cells (43). This physical barrier obviously limits the efficiency of intracranial antitumor drug delivery and intra-tumor aggregation. Numerous studies have been conducted to develop novel strategies for delivering therapeutics across the BBB, including focused ultrasound (FUS) (44) and nanotherapeutic drug delivery systems (NDDS) (45). Therefore, priorities in future research should be put on the immune access of the CNS −BBB and new ways to enhance the delivery efficiency and intra-tumor aggregation of immunotherapeutic drugs and to improve the effectiveness of GBM immunotherapy.

With the negative results reported in the CheckMate 143 trial in nivolumab-treated patients with first recurrence of GBM (46), many observers have been convinced that GBM is an immunologically typical “cold tumor”, characterized by low T-cell infiltration. To investigate the immunobiology of GBM in the clinical setting, Hao C et al. (47) found that immunohistochemistry in GBM showed sparse T lymphocyte infiltrates and abundant microglia. This phenomenon suggests an “immunosuppressed state” in GBM and may be the reason for the therapeutic failure of immunotherapy in such tumors. The immunosuppressive properties of the GBM TME significantly inhibits T-cell infiltration and activation, and such a unique microenvironment stimulates tumor cell growth and invasion. On the one hand, immunosuppressive factors, such as PD-1 and indoleamine 2,3-dioxygenase (IDO), are highly expressed in glioma cells, which limit antigen presentation. Gliomas express immunosuppressive ligands on the cell surface, including the co-stimulatory molecule B7-homolog 1 (B7-H1), also known as programmed death ligand 1 (PD-L1) (48). Loss of the tumor suppressor phosphatase and tensin homolog (PTEN) enhances phosphoinositide-3 kinase (PI3K) activity and increases surface expression of B7-H1 in glioma cells (49). This ligand can bind to and stimulate the PD-1 receptor on activated T cells resulting in T cell quiescence and apoptosis (50, 51). Several studies have shown that PD-L1 is highly expressed in GBM cells (52), and combined checkpoint blockade immunotherapy has shown good efficacy in preclinical GBM mouse models (53). However, uncertainty remains regarding the clinical efficacy of PD-1/PD-L1 checkpoint blockade in GBM. Previous studies have shown that the PD-1/PD-L1 pathway plays a role in the malignant biological behavior of GBM, but other molecular signaling networks also play an indispensable role. As demonstrated by a study of Wainwright DA et al. (19), GBM cells express IDO enzymes that can catalyze the rate-limiting step in the catabolism of tryptophan to kynurenine−a pathway that is involved in T cell immune tolerance and immunosuppression. Therefore, there is an urgent need to explore effective sub-targeted combination therapies in TME to improve the clinical response to immunotherapy for GBM.

On the other hand, the glioma microenvironment contains a large population of immunosuppressive cells, mainly including tumor-associated macrophages (TAMs) and regulatory T cells (Tregs). In GBM, TAMs include resident parenchymal microglia, perivascular macrophages and peripheral monocyte-derived cells that are recruited by GBM to release growth factors and cytokines that affect tumors (54), and TAM recruited to tumor sites can be reprogrammed by GBM cells, leading to ineffective antitumor responses (55). Past evidence suggests that context-dependent symbiotic interactions between cancer cells and TAMs are critical for GBM tumor growth through the regulation of different cytokines, chemokines, metabolites, and other factors (56). In addition, TAMs secrete interleukin-10 (IL-10) (57) and transforming growth factor β (TGF-β) (58), which reduce the activity of immune cells in the organism and provide a favorable environment for tumor growth. Tregs have been identified as a pro-tumor subpopulation of CD4+ T cells in GBM tumor tissues and the circulatory system, which can direct cytotoxic T lymphocytes (CTLs) by suppressing tumor cell immune responses (59). Moreover, gliomas exhibit a low tumor mutational burden (TML) (60) and high intra-tumor heterogeneity (61), which are also barriers to immunotherapy for GBM. Such an immune environment leads to the inability of immunotherapy to achieve similar outcomes as other tumors in the treatment of GBM. Decades of efforts to target immunotherapy for GBM have yielded limited outcomes. Further research should focus on the immune microenvironment of GBM, constantly explore new tumor-associated and tumor-specific antigens, and address the immunosuppressive properties of tumors, thus advancing future immunotherapy for GBM.

#### 4.2.3 Current status and trends of immunotherapy for GBM

There are currently more than 80 clinical trials available to evaluate the effects of immunotherapies for GBM (62), and several relevant studies have shown the potential of these immunotherapies for GBM. ICIs represent the most widely studied category of immunotherapies for GBM, including the PD-1, PDL-1 and CTLA-4 signaling pathways, which have shown promising responses in a variety of tumors (63). PD-L1 is highly expressed in GBM, making it an attractive potential target for immunotherapy trials (52). The CheckMate 143 trial was the first extensive evaluation of the efficacy of PD-1/PD-L1 immunotherapy for GBM, which assessed the efficacy of nivolumab (an anti-PD-1 monoclonal antibody) or bevacizumab in 369 patients with rGBM (64). The results showed that there was no significant difference in median OS between the two groups (9.8 months for nivolumab vs. 10.0 months for bevacizumab), but the objective response rate to treatment was higher in the bevacizumab group than that in the nivolumab group. Two recent trials, namely, CheckMate 548 trial and Checkmate 498 trial evaluated the role of nivolumab in newly diagnosed GBM. Patients received standard therapy (RT+TMZ) or standard therapy + nivolumab in Checkmate 548 trial (65), received RT+TMZ or RT + nivolumab in Checkmate 498 trial (66). The results of these two trials manifested that combination immunotherapy did not prolong the survival of GBM patients. In addition, some preclinical and clinical data show that the administration of dexamethasone alongside anti-PD-1 therapy decreases the survival of GBM patients in a dose-dependent manner (67). Dexamethasone reduces T-lymphocyte count by promoting apoptosis, in addition to decreasing lymphocyte functional capacity. Although some GBM patients are receiving long-term dexamethasone therapy for tumor invasion or radiation therapy-related brain edema, the physiological effects of steroids must be addressed in future clinical trials.

CTLA-4, also known as CD152, is a high-affinity receptor for B7 that induces negative costimulatory signaling on activated T cells (68). The CTLA-4 inhibitor, ipilimumab, is currently in clinical trials in GBM including NCT04323046, NCT04396860, NCT04817254 (69). A phase I clinical trial investigated the intracerebral (IC) administration of ipilimumab (IPI) and nivolumab (NIVO) in combination with intravenous administration of NIVO (70). The results showed that IC administration of NIVO and IPI following maximal safe resection of rGBM was feasible, safe, and associated with a prolonged OS. It is evident that ICIs hold great promise in the treatment of primary and recurrent brain tumors and treatment-induced immune-related adverse events (IrAEs) (71). An interim result reported from a phase I/II clinical trial [NCT03174197] of the PD-L1 antagonist atezolizumab with TMZ and RT showed that more than half of the patients enrolled had Grade 3 or higher adverse events that were likely related to treatment (72). In the future, increased awareness of the risks associated with ICIs and combination therapies will be essential to support treatment decisions.

Cancer vaccines work by exposing tumor-associated antigens to antigen-presenting cells (APCs), which activate immune effector cells to achieve an anti-cancer immune response. GBM-specific targets are scarce, but several targets have been identified that are specifically expressed or abundant in tumor cells, including EGFRvIII, which is a mutant version of the EGFR receptor. The cytomegalovirus (CMV) tegument phosphoprotein 65 (pp65) and IDH1 (R132H)-mutant peptides are frequently and specifically expressed in GBM (73–75). Rindopepimut (CDX-110) is a peptide vaccine that targets EGFRvIII, consisting of a unique EGFRvIII peptide sequence conjugated to keyhole limpet hemocyanin that serves as an adjuvant and activates both the humoral and cellular immunity (76). The phase II trial (36) and phase III trial (77) on EGFRvIII-targeted peptide vaccine showed improved PFS and OS compared to historical cohorts, and patients could be safely treated with Rindopepimut for a longer period. The results of the recently reported double-blind randomized phase II trial of Rindopepimut with Bevacizumab for patients with relapsed EGFRvIII-expressing glioblastoma (ReACT) showed longer OS and better overall response rates in patients receiving the peptide vaccine compared with placebo (78), suggesting a possible synergy between Rindopepimut with Bevacizumab. In future studies, the therapeutic strategy that combines Rindopepimut with other immunotherapies to improve the efficacy of GBM can continue to attract wide attention. In addition, multi-targeted vaccines that initiate immune responses to multiple tumor-associated antigens may better address intra-tumor heterogeneity.

Adoptive cell therapy (ACT) is a highly personalized cancer therapy that involves the administration of immune cells with direct anti-cancer activity to a cancer-bearing host (79). Autologous CAR T-cell therapy was the first ACT therapy to enter clinical translation and commercialization, achieving significant improvements in patients with invasive B-cell malignancies. CAR T-cell therapy is a novel treatment modality that exploits the patient’s immune system to fight cancer (80). CAR T cells overcome the limitations of previous T cell-based immunotherapies by redirecting T cell responses to specific tumor antigens (81). First-generation CAR T cells, only containing a single CD3 ζ-signaling module, show poor proliferative responses and low cytotoxicity, resulting in poor antitumor efficacy (82). The currently approved CAR T-cell therapies are second-generation CARs containing CD28 or 4-1BB signaling domains (83). The design of the CAR structure affects the pharmacokinetic profile of CAR T cells, thereby affecting the efficacy and the likelihood of adverse events. Future research will focus on the development of next-generation CAR with the aim of improving the efficacy and safety of CAR T cells. Following the success of the paradigm shift in CAR-engineered adoptive T cell therapies and advances in technologies that can transform cells into powerful antitumor weapons, the interest in NK cells as immunotherapy candidates has grown exponentially (84). T-cell receptors (TCRs) guide NK-92 cells, which have recently been shown to mediate successful antitumor responses (85).

Of all ACT-based immunotherapies currently in development for GBM, genetically engineered CAR T cells are at the forefront, with encouraging results reported in several clinical trials (86). O’Rourke et al. (34) reported 10 rGBM patients treated with a single dose of intravenous second-generation (i.e., 4-1BB, CD3ζ) EGFRvIII CAR T-cell therapy [NCT02209376] and found that the manufacture and infusion of CAR-modified T-cell (CART)-EGFRvIII cells were feasible and safe, without evidence of extratumoral toxicity or cytokine release syndrome. Ahmed N et al. (87) reported an open-label phase I dose-escalation trial, which demonstrated that the infusion of autologous HER2-specific CAR-modified virus-specific T cells (HER2-CAR VSTs) is safe and may be associated with clinical benefit in progressive GBM patients. Moreover, further evaluation of HER2-CAR VSTs as a single agent or in combination with other immunomodulatory methods for the treatment of GBM is warranted in phase 2b study. In addition, the prospect of combining ACT with other treatments for GBM currently under investigation, such as immune checkpoint blockade or oncolytic viruses, has been demonstrated (88, 89). CAR technology has emerged as a particularly attractive area of research related to GBM (90). The clinical trial results of ACT immunotherapy, particularly with CAR T cells, demonstrate a safe and feasible strategy for eliciting an effective immune response in GBM as well as great potential. However, ACT immunotherapy still faces many challenges before the full potential of ACT can be realized, and the need for continued familiarity with ACT in the future may contribute to a deeper understanding of the general mechanisms of cellular immunity and its role in GBM.

Engineered viruses constitute a promising therapeutic approach to addressing the immunosuppression of the GBM microenvironment by killing tumor cells through direct lysis and stimulation of anti-tumor immune responses (91). The most common viruses are herpesviruses, reoviruses, pox virus, or adenoviruses, which are subjected to varying degrees of genetic engineering (91, 92). It has been previously demonstrated that Zika virus (ZIKV) targets GBM stem cells and prevents death of mice with gliomas. In addition, Zika virus can be used to target GBM tissues, generating an immune-sensitized ZIKV strain that is effective alone or in combination with immunotherapy (93, 94). Hence, oncolytic ZIKV treatment can be adopted by immunotherapies, which may facilitate combination therapy for GBM. Desjardins A et al. (95) demonstrated that intratumoral infusion of PVSRIPO in rGBM patients had no neuroviral potential, and that patients treated with PVSRIPO had higher survival rates than historical controls at 24 months and 36 months. DNX-2401 (Delta-24-RGD; tasadenoturev) is a tumor-selective, replication-competent oncolytic adenovirus. Lang FF et al. (25) reported that treatment with DNX-2401 resulted in dramatic responses with long-term survival in recurrent high-grade gliomas, possibly due to the direct tumorolytic effect of the virus, which then stimulates an immune-mediated anti-glioma response. teserpaturev, as one of the genetically engineered oncolytic viruses (OVs) based on herpes simplex virus-1 (G47Δ), is the first oncolytic virus approved for the treatment of malignant gliomas (96). Several other clinical trials on the lysis of viral therapy for GBM are currently underway (97), and attention should be paid to the results of these trial data, as it is crucial to understand the current status of viral therapy for GBM. Several completed trials have demonstrated that OVs therapy is a safe and promising treatment modality for GBM patients, and further optimization of drug delivery and exploration of multimodal combination therapy options are needed to fully realize its therapeutic potential in the future.

Furthermore, gene therapy (98), TAM therapy (99), and recombinant cytokines such as IL-10 (51), interferon transforming growth factor-β (TGF-β) (100), colony- stimulating factor 1 receptor (CSF1R) (62) have been used in GBM clinical trials and have shown some therapeutic potential in specific glioma populations. As research delves deeper and data accumulate, more meaningful advances in immunotherapy regimens for gliomas will be achieved, but many obstacles and difficulties still need to be overcome before these regimens can be used in the clinic, especially difficulties in drug delivery, immune heterogeneity, and tumor heterogeneity. Additionally, the combination strategy of multiple immunotherapies, or the combination strategy of immunotherapy with targeted therapy and chemotherapy, may be an effective solution to these problems, which is worthy of further exploration in the future.

### 4.3 Limitations

The limitations of this study are as follows: 1) The data of this study were only obtained from WOScc, in English only, and other database sources and literature in other languages may be missing. 2) WOS literature is constantly updated, but the search time span in this study was from 2012 to July 2022. 3) Manual removal of unrelated documents from the study by the reviewer might lead to selection bias.

## 5 Conclusions

In the present study, publications were analyzed using multiple bibliometric tools to reveal the metrological characteristics of the literature related to immunotherapy for GBM. Immunotherapy for GBM still faces great challenges, but several relevant preclinical and clinical studies have shown the potential of immunotherapy for GBM. Therefore, immunotherapy is expected to become an essential component of future glioma treatment, providing new promising treatment strategies for GBM.

## Data availability statement

The original contributions presented in the study are included in the article/**Supplementary Material**. Further inquiries can be directed to the corresponding authors.

## Author contributions

KL: Investigation, Data curation, Conceptualization, Resources, Writing – original draft, Writing – review & editing. XD: Conceptualization, Writing – original draft. CC: Formal analysis, Methodology, Software, Validation, Visualization, Writing – review & editing. YY: Project administration, Supervision, Funding acquisition, Resources, Writing – review & editing.
