# Supplementary material for: Research hotspots and trend of glioblastoma immunotherapy: a bibliometric and visual analysis
Source: Front Oncol. 2024 Aug 7;14:1361530. doi: 10.3389/fonc.2024.1361530 (PMC11339877; doi:10.3389/fonc.2024.1361530)
Supplement: Supplementary file 1 [file DataSheet_1.docx]

Fig. S1A Compound annual growth rate (GAGR) of publications form 2012 to July 2022.

Fig. S1B Relative growth rate (RGR) of publications form 2012 to July 2022.

Fig. S1C Doubling time (DT) of publications form 2012 to July 2022.


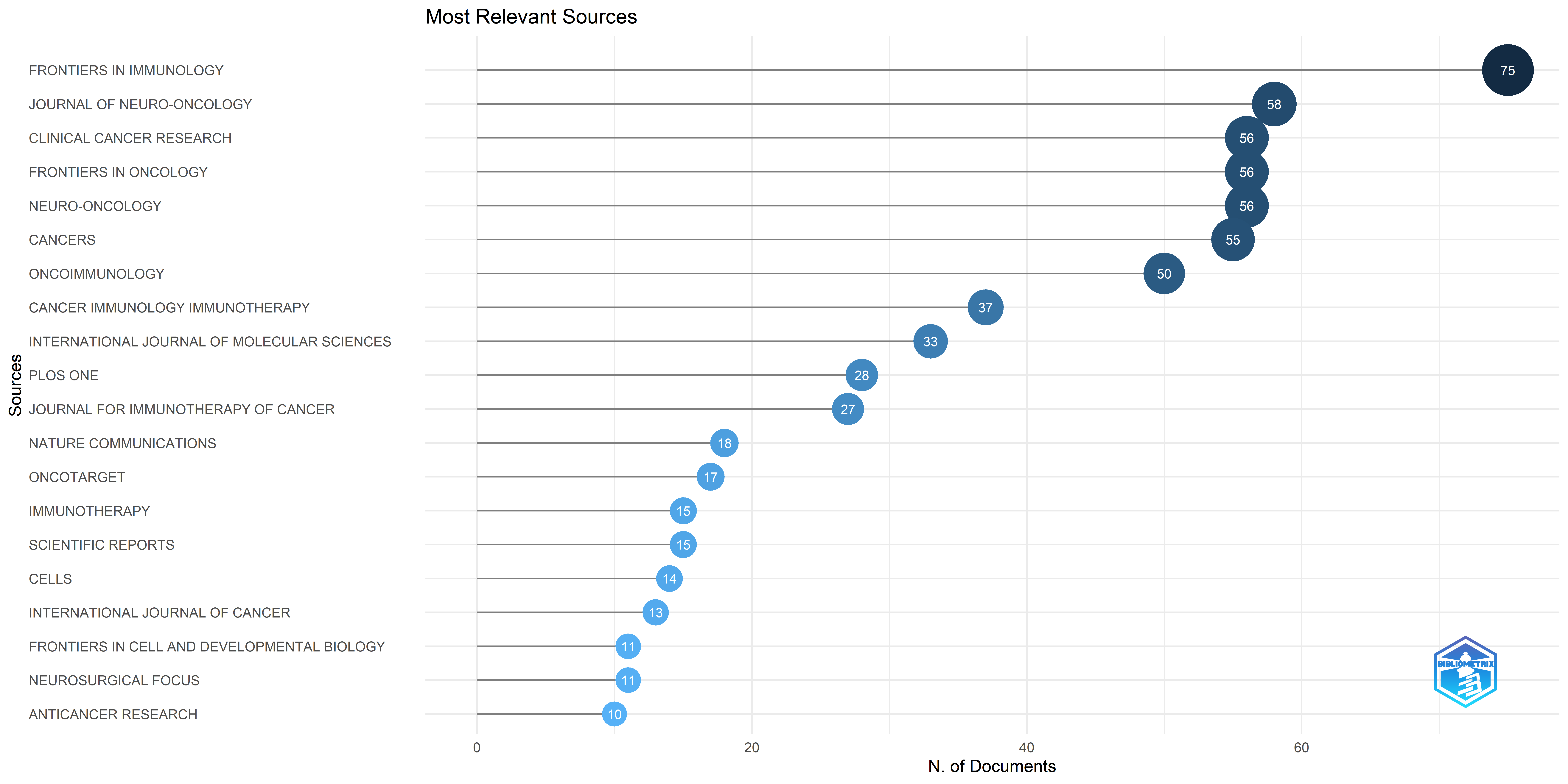


Fig. S2 The R software "bibliometrix" package for visual mapping of related publications.


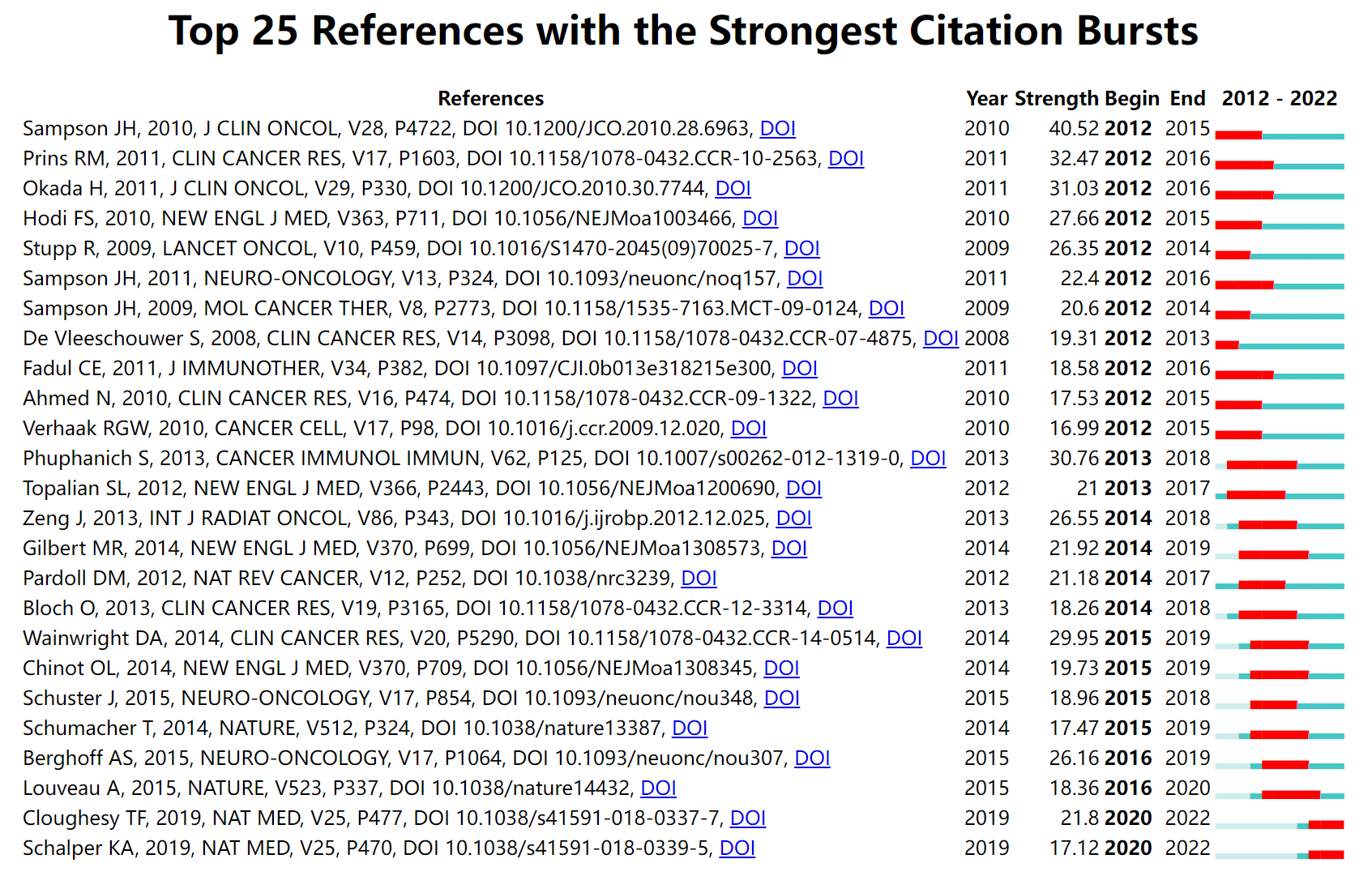


Fig. S3 CiteSpace visualization map of top 25 references with the strongest citation bursts.


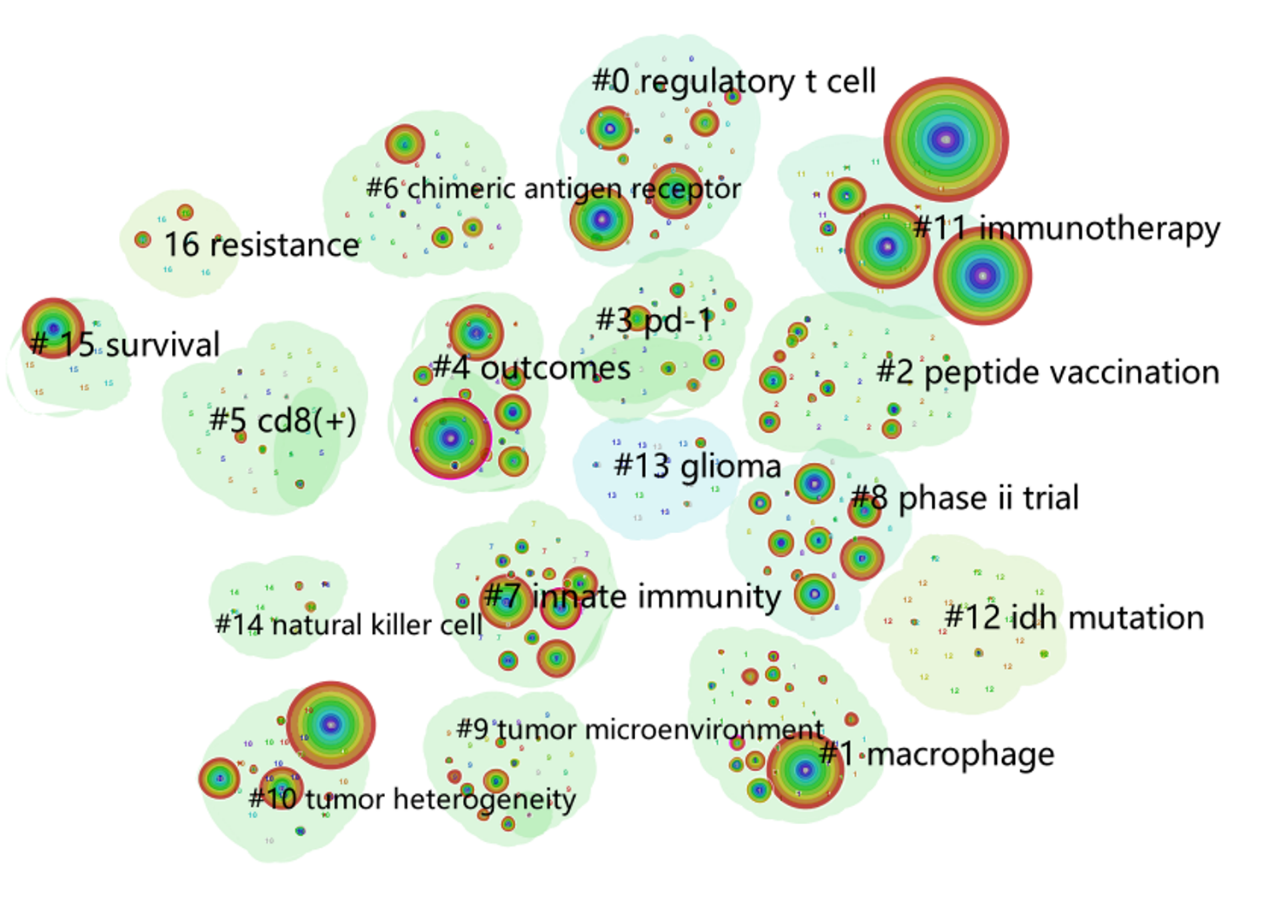


Fig. S4A CiteSpace visualization of keyword clustering mapping for related publications.


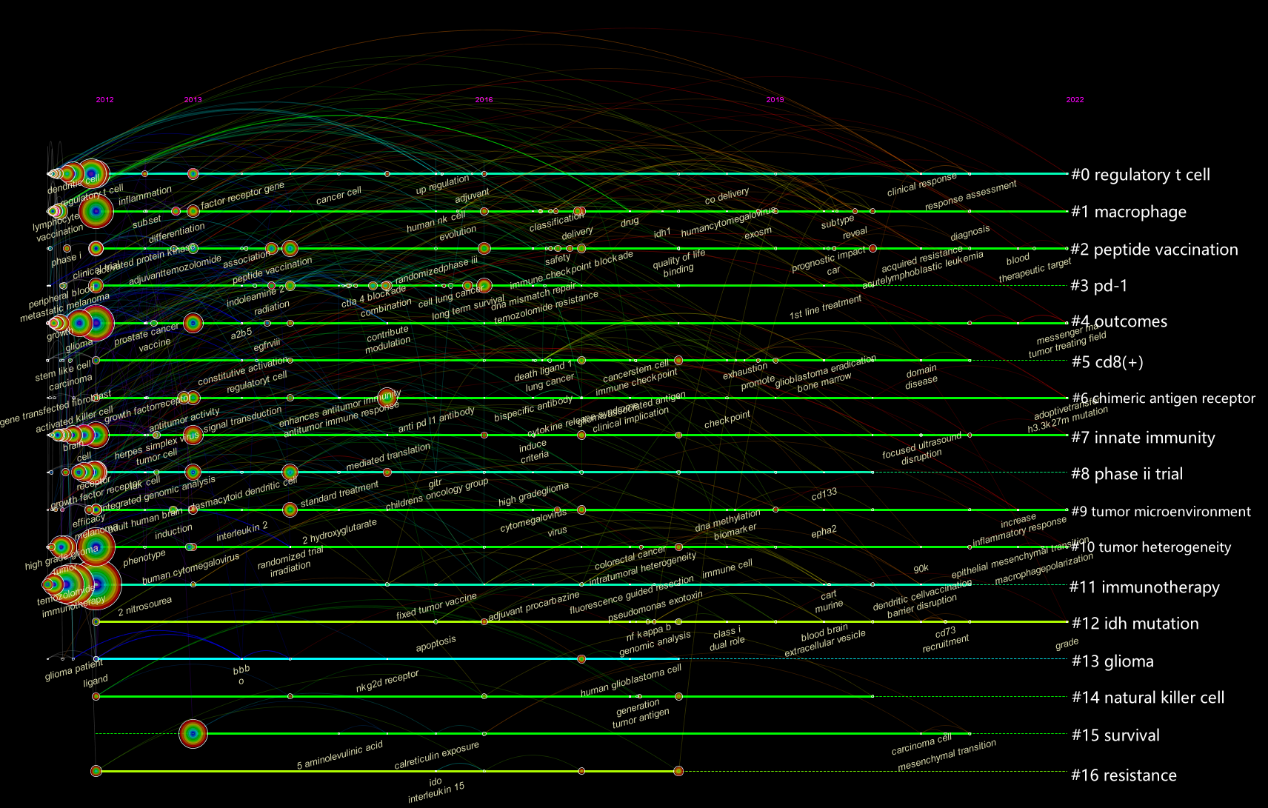


Fig. S4B Time zone map of keywords for related publications.


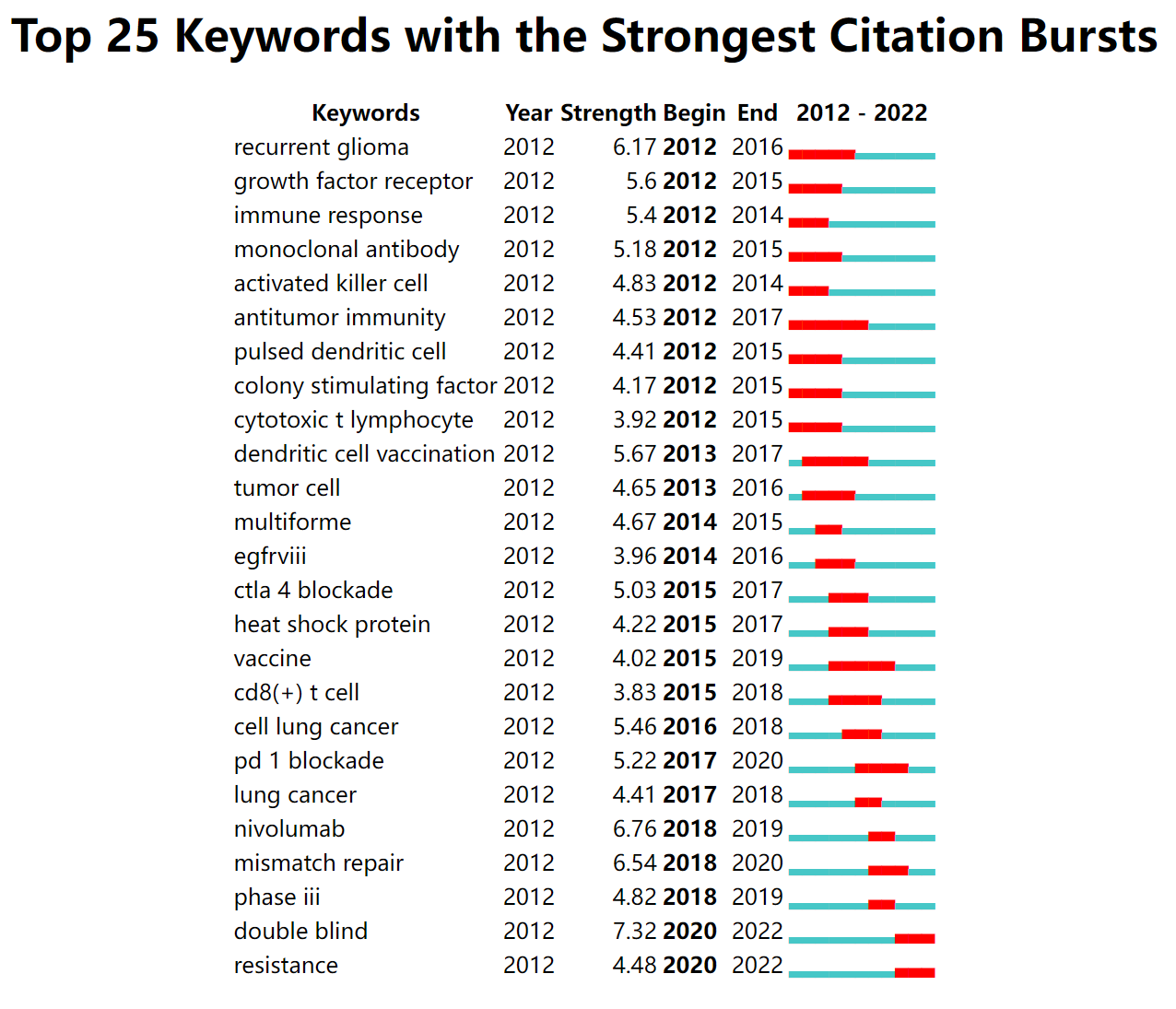


Fig.S4C Top 25 keywords with the strongest citation bursts for related publications.
