# Supplementary material for: Research hotspots and trend of glioblastoma immunotherapy: a bibliometric and visual analysis
Source: Front Oncol. 2024 Aug 7;14:1361530. doi: 10.3389/fonc.2024.1361530 (PMC11339877; doi:10.3389/fonc.2024.1361530)
Supplement: Supplementary file 2 [file DataSheet_2.docx]

Table S1. GAGR of publications from 2012 to July 2022

| Year | Number of publications | Cumarticles Total | GAGR |
| --- | --- | --- | --- |
| 2012 | 79 | 79 |  |
| 2013 | 65 | 144 | 35.01% |
| 2014 | 88 | 232 | 43.20% |
| 2015 | 80 | 312 | 40.97% |
| 2016 | 114 | 426 | 40.07% |
| 2017 | 136 | 562 | 38.68% |
| 2018 | 157 | 719 | 37.09% |
| 2019 | 165 | 884 | 35.24% |
| 2020 | 210 | 1094 | 33.91% |
| 2021 | 335 | 1429 | 33.58% |
| 2022 | 184 | 1613 | 31.35% |

Table S2. RGR and DT of publications from 2012 to July 2022

| Year | Number of publications | Cumarticles Total | RGR | DT |
| --- | --- | --- | --- | --- |
| 2012 | 79 | 79 |  |  |
| 2013 | 65 | 144 | 60.04% | 1.15 |
| 2014 | 88 | 232 | 47.69% | 1.45 |
| 2015 | 80 | 312 | 29.63% | 2.34 |
| 2016 | 114 | 426 | 31.14% | 2.23 |
| 2017 | 136 | 562 | 27.71% | 2.50 |
| 2018 | 157 | 719 | 24.64% | 2.81 |
| 2019 | 165 | 884 | 20.66% | 3.35 |
| 2020 | 210 | 1094 | 21.31% | 3.25 |
| 2021 | 335 | 1429 | 26.71% | 2.59 |
| 2022 | 184 | 1613 | 12.11% | 5.72 |

Table S3. Top 10 countries/regions for related publications

| Country | Count | Percentage | SCP | MCP | MCP-Ratio |
| --- | --- | --- | --- | --- | --- |
| USA | 717 | 44.45% | 571 | 146 | 20.36% |
| CHINA | 283 | 17.55% | 244 | 39 | 13.78% |
| GERMANY | 79 | 4.90% | 55 | 24 | 30.38% |
| ITALY | 62 | 3.84% | 56 | 6 | 9.68% |
| JAPAN | 56 | 3.47% | 51 | 5 | 8.93% |
| SWITZERLAND | 39 | 2.42% | 21 | 18 | 46.15% |
| UNITED KINGDOM | 30 | 1.86% | 18 | 12 | 40.00% |
| KOREA | 28 | 1.74% | 25 | 3 | 10.71% |
| BELGIUM | 27 | 1.67% | 16 | 11 | 40.74% |
| AUSTRALIA | 26 | 1.61% | 18 | 8 | 30.77% |

Table S4. Top 10 authors and co-cited authors in relevant publications

| Rank | Count | | Centrality | Authours | Rank | Count | Centrality | Co-cited authours |
| --- | --- | --- | --- | --- | --- | --- | --- | --- |
| 1 | | 51 | 0.1 | JOHN H SAMPSON | 1 | 805 | 0 | STUPP R |
| 2 | | 43 | 0.21 | MICHAEL LIM | 2 | 448 | 0 | REARDON DA |
| 3 | | 29 | 0.06 | DAVID A REARDON | 3 | 432 | 0.01 | SAMPSON JH |
| 4 | | 27 | 0.06 | DUANE A MITCHELL | 4 | 330 | 0 | WELLER M |
| 5 | | 25 | 0.09 | HIDEHO OKADA | 5 | 315 | 0.05 | OSTROM QT |
| 6 | | 22 | 0.18 | AMY B HEIMBERGER | 6 | 304 | 0.02 | BROWN CE |
| 7 | | 22 | 0.02 | MICHAEL WELLER | 7 | 288 | 0 | LOUIS DN |
| 8 | | 21 | 0.09 | MARIA G CASTRO | 8 | 270 | 0 | WEN PY |
| 9 | | 16 | 0.04 | E ANTONIO CHIOCCA | 9 | 261 | 0.1 | FECCI PE |
| 10 | | 15 | 0 | PEDRO R LOWENSTEIN | 10 | 238 | 0.03 | LIAU LM |

Table S5. Top 10 keywords for related publications

| Rank | Count | Centrality | Keywords |
| --- | --- | --- | --- |
| 1 | 672 | 0.01 | glioblastoma |
| 2 | 377 | 0 | immunotherapy |
| 3 | 322 | 0 | expression |
| 4 | 273 | 0.02 | temozolomide |
| 5 | 261 | 0.12 | cancer |
| 6 | 212 | 0.01 | t cell |
| 7 | 178 | 0.06 | survival |
| 8 | 173 | 0.02 | glioma |
| 9 | 167 | 0.01 | central nervous system |
| 10 | 152 | 0 | regulatory t cell |
